# Cloaking a qubit in a cavity

**DOI:** 10.1038/s41467-023-42060-5

**Published:** 2023-10-09

**Authors:** Cristóbal Lledó, Rémy Dassonneville, Adrien Moulinas, Joachim Cohen, Ross Shillito, Audrey Bienfait, Benjamin Huard, Alexandre Blais

**Affiliations:** 1 Institut Quantique and Département de Physique, Université de Sherbrooke, Sherbrooke, J1K 2R1 QC Canada; 2 Ecole Normale Supérieure de Lyon, CNRS, Laboratoire de Physique, F-69342 Lyon, France; 3 Canadian Institute for Advanced Research, Toronto, ON M5G1M1 Canada

**Keywords:** Qubits, Single photons and quantum effects

## Abstract

Cavity quantum electrodynamics (QED) uses a cavity to engineer the mode structure of the vacuum electromagnetic field such as to enhance the interaction between light and matter. Exploiting these ideas in solid-state systems has lead to circuit QED which has emerged as a valuable tool to explore the rich physics of quantum optics and as a platform for quantum computation. Here we introduce a simple approach to further engineer the light-matter interaction in a driven cavity by controllably decoupling a qubit from the cavity’s photon population, effectively cloaking the qubit from the cavity. This is realized by driving the qubit with an external tone tailored to destructively interfere with the cavity field, leaving the qubit to interact with a cavity which appears to be in the vacuum state. Our experiment demonstrates how qubit cloaking can be exploited to cancel the ac-Stark shift and measurement-induced dephasing, and to accelerate qubit readout. In addition to qubit readout, applications of this method include qubit logical operations and the preparation of non-classical cavity states in circuit QED and other cavity-based setups.

## Abstract (web summary)

Circuit QED with strongly driven cavities is a powerful framework for quantum technologies, but often undesired effects on the qubit are introduced. Here, by using an external tone tailored to destructively interfere with the cavity field, the authors show how a transmon can be protected from these unwanted effects.

## Introduction

Cavity and circuit QED explore light–matter interaction at its most fundamental level, providing the tools to control the dynamical evolution of single atoms and photons in a deterministic fashion^1^. This has allowed circuit QED to emerge as a platform to explore the rich physics of quantum optics in novel parameter regimes and to become a leading architecture for quantum computing^2^. In this system, strong drives on the cavity are used to realize multi-qubit gates^3,4^, to stabilize quantum states of the cavity^5–8^, and for qubit readout^2,9^. However, even under moderate cavity photon populations, cavity drives often lead to undesired effects such as qubit transitions^10^ resulting in reduced readout fidelity^11^, increased dephasing^4,7^, and imperfect quantum state stabilization^5,6,8^. Other consequences of cavity drives in the dispersive qubit-cavity regime are the qubit ac-Stark shift, which can result in unwanted phase accumulations^12^, measurement-induced dephasing^13,14^, and Kerr nonlinearity^15^.

**Fig. 1:**
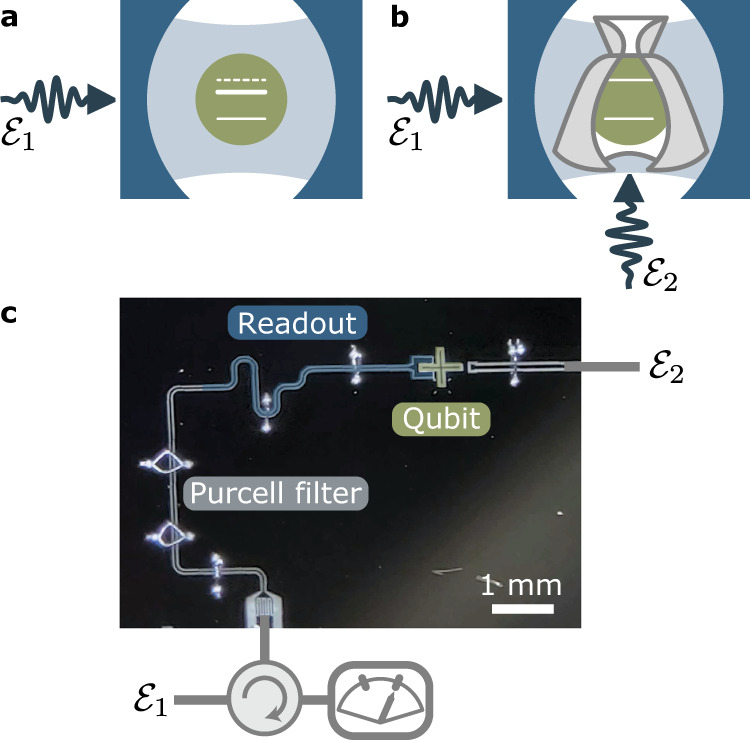
Concept and device. **a** Schematic illustration of a qubit (green) coupled to a driven cavity represented by two mirrors (blue). A drive ${{{{{{{{\mathcal{E}}}}}}}}}_{1}$ on the cavity displaces the cavity field, which effectively acts as a classical drive on the qubit. This results in qubit ac-Stark shift and measurement-induced dephasing (i.e. level broadening), see the full white lines. **b** A second drive ${{{{{{{{\mathcal{E}}}}}}}}}_{2}$ of appropriate time-dependent amplitude, frequency, and phase cloaks the qubit from the effective classical field resulting from ${{{{{{{{\mathcal{E}}}}}}}}}_{1}$ interferometrically canceling the ac-Stark shift and broadening. **c** Optical image of the device which includes a transmon qubit (green), a readout cavity (dark blue), and a Purcell filter (gray).

In this work, we introduce a simple approach to engineer light–matter interaction in a driven cavity and prevent some of these unwanted effects. We show how an appropriately tailored drive on the qubit can decouple the qubit state and the cavity’s photon population from one another, resulting in both systems interacting only through vacuum fluctuations of the cavity field. This qubit cloaking mechanism can be exploited to prepare non-classical states of the cavity field. Moreover, in the dispersive qubit–cavity regime, it results in the absence of ac-Stark shift and measurement-induced dephasing. This observation can be used to apply logical operations on the qubit in the presence of a cavity photon population, something which we exploit to accelerate qubit readout. Here, we experimentally demonstrate qubit cloaking using a transmon qubit^16^ coupled to a coplanar waveguide resonator (Fig. 1c).

## Results

When loaded with a coherent state, the cavity field acts as an effective classical drive on the qubit. A simple intuition behind qubit cloaking is that an additional drive on the qubit can be designed to interfere destructively with this effective drive, resulting in an empty cavity from the perspective of the qubit. As an illustration of this concept, consider a transmon qubit coupled to a microwave cavity, see Fig. 1a, b for a schematic representation. Without driving, the system Hamiltonian reads^2^

1 $${\hat{H}}_{0}=4{E}_{\rm {{C}}}{\hat{n}}_{{{{{{{{\rm{tr}}}}}}}}}^{2}-{E}_{\rm {{J}}}\cos {\hat{\varphi }}_{{{{{{{{\rm{tr}}}}}}}}}+\hslash {\omega }_{\rm {{r}}}{\hat{a}}^{{{{\dagger}}} }\hat{a}+i\hslash g{\hat{n}}_{{{{{{{{\rm{tr}}}}}}}}}({\hat{a}}^{{{{\dagger}}} }-\hat{a}),$$

with ${\hat{n}}_{{{{{{{{\rm{tr}}}}}}}}}$ and ${\hat{\varphi }}_{{{{{{{{\rm{tr}}}}}}}}}$ the transmon charge and phase operators, *E*_C_ the charging energy, *E*_J_ the Josephson energy, ${\hat{a}}^{({{{\dagger}}} )}$ the annihilation (creation) operator of the cavity mode of frequency *ω*_r_, and *g* the transverse coupling rate. The cavity drive is ${\hat{H}}_{1}=i\hslash {{{{{{{{\mathcal{E}}}}}}}}}_{1}(t)({\hat{a}}^{{{{\dagger}}} }-\hat{a})$, where ${{{{{{{{\mathcal{E}}}}}}}}}_{1}(t)={\varepsilon }_{1}(t)\sin ({\omega }_{1}t+{\phi }_{1})$ is the amplitude of the cavity drive at frequency *ω*_1_ with envelope *ε*_1_(*t*). The cloaking is triggered by a canceling drive ${{{{{{{{\mathcal{E}}}}}}}}}_{2}(t)$ on the transmon qubit, which leads to an additional term ${\hat{H}}_{2}=\hslash {{{{{{{{\mathcal{E}}}}}}}}}_{2}(t){\hat{n}}_{{{{{{{{\rm{tr}}}}}}}}}$, such that the total system Hamiltonian is $\hat{H}={\hat{H}}_{0}+{\hat{H}}_{1}+{\hat{H}}_{2}$.

The appropriate choice of ${{{{{{{{\mathcal{E}}}}}}}}}_{2}(t)$ that cloaks the qubit from the cavity field is revealed by moving to a displaced frame using the transformation $\hat{D}({\alpha }_{t})=\exp ({\alpha }_{t}{\hat{a}}^{{{{\dagger}}} }-{\alpha }_{t}^{*}\hat{a})$ under which ${\hat{D}}^{{{{\dagger}}} }({\alpha }_{t})\hat{a}\hat{D}({\alpha }_{t})=\hat{a}+{\alpha }_{t}$. Choosing ${\alpha }_{t}=\int\nolimits_{0}^{t}{{{{{\rm{d}}}}}}\tau {{{{{{{{\mathcal{E}}}}}}}}}_{1}(\tau ){{{{{{\rm{e}}}}}}}^{-i{\omega }_{r}(t-\tau )}$ has the effect of canceling the cavity drive ${\hat{H}}_{1}$ in the displaced Hamiltonian, resulting in an effective drive on the qubit of the form $ig({\alpha }_{t}^{*}-{\alpha }_{t}){\hat{n}}_{{{{{{{{\rm{tr}}}}}}}}}$ owing to the qubit–cavity coupling. Therefore, taking the amplitude of the canceling tone to be the opposite

2 $${{{{{{{{\mathcal{E}}}}}}}}}_{2}(t)=-ig\left[{\alpha }_{t}^{*}-{\alpha }_{t}\right],$$

disables any drive term in the displaced frame, where the total Hamiltonian comes down to (see Supplementary Note 1 for more details)

3 $${\hat{H}}^{{\prime} }={\hat{D}}^{{{{\dagger}}} }({\alpha }_{t})\hat{H}\hat{D}({\alpha }_{t})-i{\hat{D}}^{{{{\dagger}}} }({\alpha }_{t})\dot{\hat{D}}({\alpha }_{t})={\hat{H}}_{0}.$$

In short, despite the presence of the drive ${{{{{{{{\mathcal{E}}}}}}}}}_{1}$ populating the cavity with an average photon number ∣*α*_*t*_∣^2^, the qubit experiences the cavity as if it were in the vacuum state, only coupling to vacuum fluctuations of the cavity field. Note that no approximations have been made to arrive at this result which is valid irrespective of the qubit–cavity detuning and for arbitrary time-dependent drive amplitude *ε*_1_(*t*) and frequency *ω*_1_. Beyond the transmon qubit, this result is valid for two-level systems and any nonlinear system linearly coupled to a cavity. The results are unchanged in the presence of qubit decay or dephasing, and the above derivation can exactly account for the finite decay rate *κ* of the cavity by making the change *ω*_r_ → *ω*_r_ − *iκ*/2 in the expression for *α*_*t*_. In the rotating-wave approximation and for a constant *ε*_1_, the amplitude *α*_*t*_ entering the expression for ${{{{{{{{\mathcal{E}}}}}}}}}_{2}$ in Eq. (2) is $({\varepsilon }_{1}{{{{{{\rm{e}}}}}}}^{-i({\omega }_{1}t+{\phi }_{1})}/2)/({\omega }_{\rm {{r}}}-{\omega }_{1}-i\kappa /2)$ in steady state. See Supplementary Note 1 for details.

This approach relies on two distinct driving ports—one port dedicated to exciting the qubit or nonlinear mode and a second port for driving the cavity mode—and is directly applicable in several cavity-based platforms including semiconducting quantum dots coupled to microwave resonators^17–20^, electrons on solid neon^21^, circuit quantum acoustodynamics^22^, and trapped atoms in cavity QED^23^. While it is an exact and robust result, potential limitations of qubit cloaking are discussed in Supplemental Note 3.

**Fig. 2:**
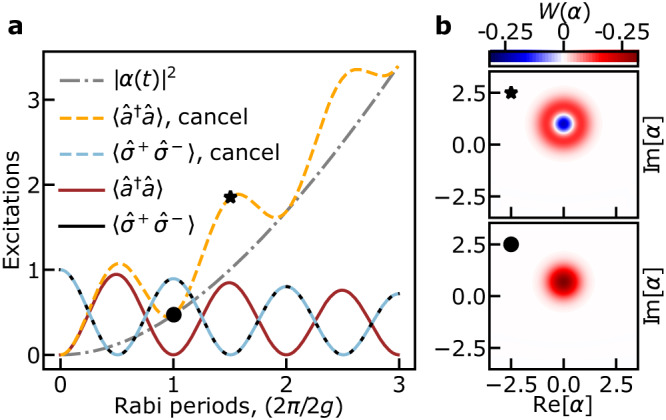
Vacuum Rabi oscillations in a filled cavity. **a** Red and black solid lines correspond to damped vacuum Rabi oscillations of the resonant cavity and qubit, respectively, in the absence of any drive. The qubit–cavity Jaynes–Cummings coupling rate ${g}^{{\prime} }$ is set to 100/7 of the cavity decay rate *κ*. With a cavity drive *ϵ*_1_ = 46*κ*/7 and the cancellation turned on, the cavity field (dashed orange) oscillates on top of ∣*α*_*t*_∣^2^ (dashed-dotted gray), swapping a single quantum of excitation back and forth with the qubit (dashed light blue). **b** Computed Wigner distributions of the cavity field at 3/2 and 1 Rabi periods with the drives on, as indicated by the symbols in (**a**). At 1 Rabi period, the cavity is in a coherent state, while it is in a displaced Fock state at 3/2 Rabi periods.

As a first example, we consider a situation where the qubit is resonant with the cavity and model the transmon as a two-level system. In the absence of any drives, initializing the qubit in its first excited state and the cavity in the vacuum state leads to vacuum Rabi oscillations at the frequency $2{g}^{{\prime} }$^1^, where ${g}^{{\prime} }=(g/2){({E}_{{{{{{\rm{J}}}}}}}/2{E}_{{{{{{\rm{C}}}}}}})}^{1/4}$ is the Jaynes–Cummings coupling^2^. This is illustrated in Fig. 2a which shows the results (full black and red lines) of integration of the system’s master equation including cavity decay, see Supplementary Note 1 for details. With the cavity drive and the canceling tone present, the cavity population (orange dashed lines) increases following ∣*α*_*t*_∣^2^ (dashed-dotted gray line) on top of which oscillations are observed. On the other hand, the qubit population (light blue dashed line) is identical to that observed in the absence of the drives. In other words, instead of collapse and revival which are expected in the presence of a coherent state in the cavity^24,25^, here the qubit undergoes oscillations at the vacuum Rabi frequency. The same conclusion holds when initializing the cavity mode in the Fock state $\left|1\right\rangle$ and the qubit in the ground state (not shown). This is a clear illustration that under cloaking the qubit only couples to vacuum fluctuations of the cavity. In related work, Alsing et al.^26^ have shown how a drive on the atom can suppress atomic fluorescence in the steady-state of a lossless cavity. In contrast, cloaking is an exact result that is valid at all times and in the presence of loss. Moreover, this approach can be used to prepare non-classical states of the cavity field. For example, displaced Fock states $\hat{D}(\alpha )\left|n=1\right\rangle$ with arbitrary *α* can be prepared by starting with the same initial state as in Fig. 2a, and waiting for half-integer Rabi periods under appropriate cavity drive amplitude and phase. The Wigner function of the displaced Fock state is plotted at 3/2 Rabi periods in Fig. 2b and is compared to that of the coherent state at 1 Rabi period.

**Fig. 3:**
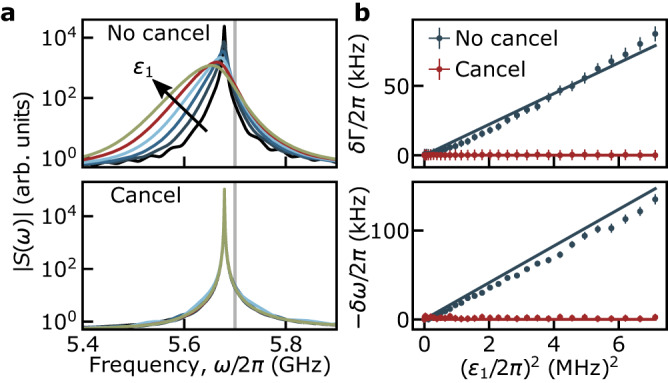
Cancellation of the ac-Stark shift and measurement-induced dephasing. **a** Numerical simulation of a two-level qubit spectral density without cancellation (top) and with cancellation (bottom) for various drive amplitudes *ε*_1_/2*π* from 10 to 60 MHz. The simulation parameters are *ω*_*q*_/2*π* = 5.7 GHz, *ω*_*r*,1_/2*π* = 7.6 GHz, ${g}^{{\prime} }/2\pi=200$ MHz, and *κ*/2*π* = 50 MHz. **b** Dots: Experimentally measured increased dephasing rate *δ*Γ (top) and qubit frequency shift *δω* (bottom) as a function of drive power extracted from Ramsey interferometry without cancellation (blue) and with cancellation (red). Error bars are statistical. Solid lines: Numerical simulations of the measurement-induced dephasing and ac-Stark shift. The simulation which accounts exactly for the $\cos {\hat{\varphi }}_{{{{{{{{\rm{tr}}}}}}}}}$ potential of the Josephson junction is performed using the bare parameters *ω*_*r*_/2*π* = 7.66 GHz, *E*_J_/*h* = 16.83 GHz, *E*_C_/*h* = 199.7 MHz, *g*/2*π* = 140.6 MHz, and *κ*/2*π* = 10.1 MHz resulting in dressed parameters that match the experimentally measured ones. The cavity drive frequency is *ω*_1_/2*π* = 7.6648 GHz.

As a second example, consider the typical situation of a dispersive qubit readout where the frequency of the first drive is resonant with the cavity and the qubit–cavity detuning is large compared to ${g}^{{\prime} }$ such that the system is in the dispersive regime^1,2^. In the absence of the cancellation tone, the cavity drive results in ac-Stark shift and broadening of the qubit^13,14^. This is illustrated in the top panel of Fig. 3a which shows the numerically computed magnitude of the qubit’s absorption spectrum *S*(*ω*). For simplicity, this is obtained in the two-level approximation for the transmon where the absorption spectrum takes the form $S(\omega )=(1/2\pi )\int\nolimits_{-\infty }^{+\infty }{{{{{{\rm{e}}}}}}}^{i\omega t}{\langle {\hat{\sigma }}_{-}(t){\hat{\sigma }}_{+}(0)\rangle }_{{{{{{\rm{s}}}}}}}$ with the subscript s indicating that the average is taken in steady-state. The different colored lines correspond to different cavity drive amplitudes *ε*_1_. The vertical gray line indicates the bare qubit frequency. In contrast, the bottom panel of Fig. 3a shows the same quantity obtained with the cancellation tone present. In this case, the results for the different drive amplitudes collapse on each other. Because cloaking does not affect vacuum fluctuations, the Lamb shift (i.e. the offset from the bare qubit frequency) remains unchanged at all drive amplitudes. Moreover, the independence of the qubit linewidth on drive power indicates both the absence of measurement-induced dephasing and that Purcell decay remains at its zero-photon value^27,28^ under cloaking.

This prediction is experimentally tested on the device shown in Fig. 1c. It consists of a transmon qubit capacitively coupled to a *λ*/4 microwave coplanar waveguide cavity which is driven through port 1 (${{{{{{{{\mathcal{E}}}}}}}}}_{1}$) via a Purcell filter acting as a bandpass filter at the cavity frequency. The cancellation drive (${{{{{{{{\mathcal{E}}}}}}}}}_{2}$) is applied to port 2, which is weakly capacitively coupled to the transmon. The readout frequency is *ω*_1_/2*π* = 7.6648 GHz and cavity decay rate *κ*/2*π* = 10.1 MHz. The transmon qubit is capacitively coupled to the cavity, with *χ*/2*π* = −2.54 MHz the full dispersive shift^2^. The qubit has a dressed frequency ${\tilde{\omega }}_{{{{{{\rm{q}}}}}}}/2\pi=4.96216\,{{{{{{{\rm{GHz}}}}}}}}$ and coherence times *T*_1_ = 25 μs and *T*_2_ = 7.5 μs.

Using Ramsey interferometry, it is possible to determine the decoherence rate and the qubit frequency, and thus extract the increase in dephasing rate *δ*Γ and the ac-Stark shift *δω* as a function of drive strength. The blue dots in Fig. 3b are obtained in the absence of cancellation drive and show the expected linear increase with drive power^13,14^. The full lines are obtained from numerical integration of the system’s master equation including cavity loss and are used to calibrate the attenuation factor between the drive power at room temperature and ${\epsilon }_{1}^{2}$ for all experimental plots. The red dots correspond to the measured ac-Stark shift and increase in dephasing rate in the presence of the cancellation tone. As expected from the above discussion, with the properly tailored cancellation drive, they are both suppressed at all cavity drive powers. The cancellation drive follows the analytical expression Eq. (2) for ${{{{{{{{\mathcal{E}}}}}}}}}_{2}(t)$ but the attenuation and electrical delay of the line driving port 2 need to be taken into account in order to relate the complex drive amplitude at room temperature ${{{{{{{{\mathcal{E}}}}}}}}}_{2}^{{{{{{{{\rm{room}}}}}}}}}(t)$ to the driving strength ${{{{{{{{\mathcal{E}}}}}}}}}_{2}(t)=\mu {{{{{{{\rm{Re}}}}}}}}[{{{{{{\rm{e}}}}}}}^{i\phi }{{{{{{{{\mathcal{E}}}}}}}}}_{2}^{{{{{{{{\rm{room}}}}}}}}}(t)]$. The prefactor relating the two is found experimentally by varying the phase *ϕ* and amplitude *μ* to minimize ∣*δ*Γ + *iδω*∣ (see Supplementary Note 2). This technique thus provides a tool to calibrate the attenuation from room temperature to the qubit port.

The above results suggest a strategy to speed up the dispersive qubit readout. Several approaches have been explored to improve readout fidelity by speeding it up^29–35^. Here, we propose to use a two-step ‘arm and release’ approach. With the cavity in the vacuum state, the arming step consists in driving the cavity in the presence of the canceling tone. Because the qubit is uncoupled from the cavity’s classical field, this causes a displacement of the cavity field by *α*_*t*_ that is *independent* of the qubit-state. Thus during the pre-arming, the cavity can be stabilized in any coherent state without affecting the qubit. The release step can start at any time after the desired measurement photon population is reached: The cancellation tone is turned off, at which point the qubit couples to the cavity field resulting in a qubit-state *dependent* rotation of the latter in phase space under the dispersive qubit-cavity coupling. Homodyne or heterodyne detection then completes the qubit readout in the usual way^2^.

**Fig. 4:**
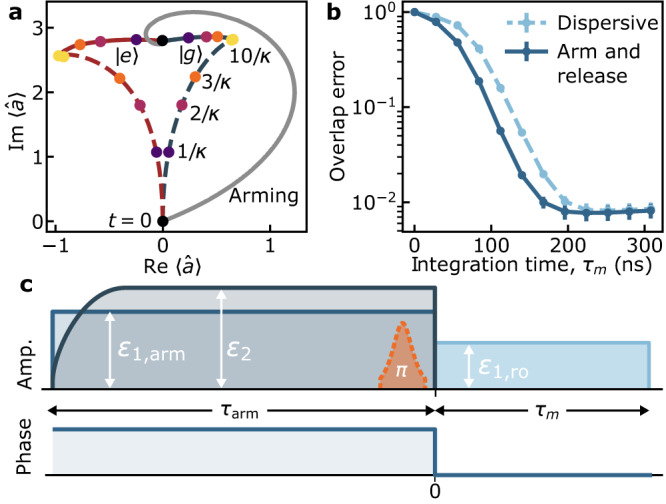
Arm and release qubit readout. **a** Full-cosine numerical simulation of the phase-space trajectories of the cavity field in standard dispersive readout, i.e., square pulse, (dashed line) and the arm and release readout approach (full lines) with an arm-step coherent state *α* = 2.8*i*. The path of the arming step is here chosen to reproduce the experiment of panel **b**, but it can be tailored at will. The colored dots indicate different times in the evolution. In both readout approaches, the qubit is prepared in the ground state and a *π* pulse is applied (red lines) or not (blue lines) before the readout step. Simulation parameters are the same as in Fig. 3b. **b** Experimental qubit measurement error obtained from the overlap between the distributions of the accumulated heterodyne signal over 10^6^ repetitions of the experiment. Full line: arm-and-release approach. Dashed line: standard dispersive measurement. **c** Arm-and-release pulse sequence used to obtain panels **a** and **b**. The arm phase (*t* < 0) is absent in the case of dispersive readout.

Crucially, upon release the two coherent states $|{\alpha }_{{{{{{\rm{g,e}}}}}}}\rangle$ corresponding to the qubit ground and excited states separate as under longitudinal coupling at short times. Indeed, as illustrated in Fig. 4a, after the arming step these coherent states move away from each other in the phase space of the cavity mode (full lines), thus maximizing the qubit measurement rate *κ*∣*α*_g_−*α*_e_∣^2^/2^14,35^. This is reminiscent of previous experiments^32,33^ where longitudinal-like separation is obtained with an initially empty cavity using an approach based on the dispersive approximation. In contrast, we emphasize that qubit cloaking can operate at arbitrary drive strength and qubit-cavity detuning. Figure 4a also shows the evolution in phase space of the amplitude of the readout cavity for the usual dispersive readout (dashed line), where there is initially poor separation between the qubit-state-dependent coherent states. Similarly to longitudinal readout^35^, for the same steady-state photon population (see yellow dots corresponding to *t* = 10/*κ*), the state separation is significantly larger at short times in the arm-and-release approach than in the usual dispersive readout (see the colored dots corresponding to times *t* = (1, 2, 3)/*κ*).

The reduction in measurement time provided by the arm-and-release approach is illustrated in Fig. 4b which shows the measurement error versus integration time for the arm-and-release approach (full line) and the standard dispersive readout (dashed line) obtained using the device of Fig. 1c. In these experiments, the qubit is first prepared in the ground state $\left|g\right\rangle$ using measurement-based feedback with the usual dispersive readout. As illustrated in Fig. 4c, to obtain the full line in panel b the cavity is then pre-armed with a drive of amplitude *ε*_1,arm_/2*π* = 95.5 MHz (full blue line) and a cancellation drive *ε*_2_ (dashed dark blue line) for a time *τ*_arm_ = 1 μs. At the time labeled *t* = 0, the signal is then integrated for a time *τ*_m_ with a cavity drive *ε*_1,ro_/2*π* = 63.7 MHz. The arming cavity drive and the cancellation tone are omitted to obtain the dashed line in panel b. A *π* pulse of width 25 ns (orange dashed line) is applied or not at the end of the arming step to prepare the state $\left|e\right\rangle$ or $\left|g\right\rangle$. Interestingly and as explained below, this can be done with high fidelity despite the cavity photon population. Finally, the measurement error is obtained by computing the overlap between the distribution of the accumulated heterodyne signal over *N* = 10^6^ repetitions of the experiment where the qubit is prepared either in $\left|g\right\rangle$ or $\left|e\right\rangle$ (see Supplementary Note 2). For a 196 ns integration time, we obtain an average fidelity ${{{{{{{\mathcal{F}}}}}}}}=1-[P({g| e})+P({e| g})]/2=(99.35\pm 0.14)\%$ where *P*(*g*∣*e*) = (1.07 ± 0.14)% is the error probability to measure state *g* when state *e* was prepared, while *P*(*e∣g*) = (0.23 ± 0.14)% is the error probability to measure state *e* when state *g* was prepared. The finite number of repetitions *N* results in the uncertainty ± 0.14%. The 0.65% average error is mostly explained by wrong preparation of the ground state before the arming step (~0.2%), imperfect *π* pulse (~0.08%), relaxation during measurement (~0.2%), and finite Gaussian separation (~0.13%).

In optimizing readout fidelity, it is important to account that the cavity responds at different frequencies in the absence or presence of the cancellation tone. Without cloaking, the qubit-state-dependent steady-states coherent state amplitude is ${\alpha }_{i}^{{{{{{{{\rm{s}}}}}}}}}=({\varepsilon }_{1}{{{{{{\rm{e}}}}}}}^{-i{\phi }_{1}}/2)({\tilde{\omega }}_{\rm {{r}}}+{\chi }_{i}-{\omega }_{1}-i\kappa /2)$, where ${\tilde{\omega }}_{\rm {{r}}}$ is the dressed cavity frequency and *χ*_*i*_ is the dispersive qubit–cavity coupling for qubit state *i* = {*g*, *e*}^2^. On the other hand, with cloaking the cavity responds as if there was no qubit with the steady-state value ${\alpha }^{{{{{{{{\rm{s}}}}}}}}}=({\varepsilon }_{1}{{{{{{\rm{e}}}}}}}^{-i{\phi }_{1}}/2)/({\omega }_{{{{{{\rm{r}}}}}}}-{\omega }_{1}-i\kappa /2)$, where now *ω*_r_ is the bare cavity frequency. Depending on the application, such as optimizing the longitudinal-like nature of the readout, an optimal coherent state can be prepared by adequately choosing *ε*_1_ and *ϕ*_1_ during the arming phase, and changing these quantities as desired during the release phase; see Fig. 4c for the pulse sequence and phase used here for readout. The arming phase-space path shown in Fig. 4a is chosen to replicate our experiment, but it can be tailored. For example, a straight path is obtained by arming the cavity with a drive frequency *ω*_1_ = *ω*_r_.

**Fig. 5:**
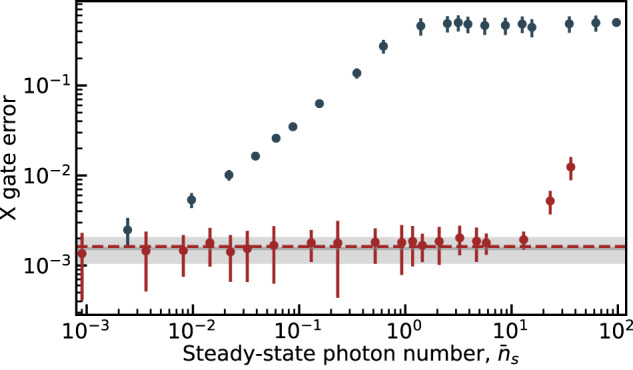
Gate error. Measured error (dots) on qubit *X* gate (randomized benchmarking) as a function of the steady-state cavity photon number ${\bar{n}}_{{{{{{{{\rm{s}}}}}}}}}$ when the qubit is cloaked (red) or not (blue). The control drive on the qubit is optimized in the absence of cavity drive and cancellation tone to maximize the gate fidelity and kept identical for all further measurements. The corresponding measured average *X* gate error and its error bar at zero cavity drive amplitude are represented as a horizontal gray line and gray area. The dashed red line is the average gate error under cloaking obtained from numerical simulations including the Purcell filter (see Supplementary Note 1). Error bars account for statistical uncertainty.

As an additional benefit, since the cloaked qubit does not undergo ac-Stark shift or measurement-induced dephasing (see Fig. 3), it is possible to apply qubit gates during the cavity arming step despite the presence of measurement photons in the cavity. As a result, the time needed to fill the cavity does not factor in the measurement time. To test this idea, we use randomized benchmarking^36,37^ on the device of Fig. 1c and extract the gate error for a qubit *π*-pulse (X gate) performed in the presence of a readout cavity drive, and in the absence or presence of the cancellation tone, see Fig. 5. As expected, in the absence of cancellation (blue dots) the gate error increases rapidly with the cavity drive amplitude and saturates at a gate error of 0.5, corresponding to the largest gate error which can be reported by randomized benchmarking^38^. In the presence of cancellation (red dots), the gate error remains at its coherence-limited value (full gray line) despite the presence of measurement photons in the readout cavity. The red dashed line corresponds to the result of numerical simulations accounting for the Purcell filter of the gate under cloaking (see Supplementary Note 1). Importantly, the qubit control drive is identical for all data points shown in Fig. 5. For large drive amplitudes, the X gate error under cloaking starts to increase (last three points in Fig. 5). This is explained by imperfect experimental calibration of the cancellation tone which can be affected by low-frequency drifts.

## Discussion

Qubit cloaking can readily be implemented in current circuit QED experiments. This approach is valid irrespective of the driving frequency and waveshape, and applies to arbitrary qubit–cavity detuning. In the resonant regime, cloaking can be used to prepare non-classical states of the cavity^39,40^. In the dispersive regime, cloaking can be used to speed-up qubit readout^41^ something which, for example, can be used to shorten quantum error correction cycles in circuit QED-based devices^42–44^. At the heart of this acceleration is the fact that logical gates can be applied to the qubit while the cavity is armed for readout. Similar ideas can be exploited to speed up two-qubit gates that are assisted by a cavity drive, such as the resonator-induced phase gate^3^, or to reduce measurement cross-talk errors in multiplexed readout^45^. Moreover, the possibility to cancel large ac-Stark shifts can be leveraged to avoid unwanted phase accumulations, such as in the preparation of bosonic codes states^12^. We expect that qubit cloaking will become a useful element in the toolbox of circuit QED, extending beyond the transmon qubit^46^. Furthermore, it is applicable to other cavity QED setups where a quantum system linearly couples to a cavity^17,19,20,22,23,47^.

### Supplementary information

Supplementary Information

Peer Review File

## Data Availability

The data generated in this study have been deposited in the Figshare database^48^.
