# Supplementary material for: Cloaking a qubit in a cavity
Source: Nat Commun. 2023 Oct 9;14:6313. doi: 10.1038/s41467-023-42060-5 (PMC10562410; doi:10.1038/s41467-023-42060-5)
Supplement: Supplementary file 1 — Supplementary Information [file 41467_2023_42060_MOESM1_ESM.pdf]

Supplementary Information for Cloaking a qubit in a cavity

Cristóbal Lledó*,[1] Rémy Dassonneville*,[2] Adrien Moulinas,[1] Joachim Cohen,[1]
Ross Shillito,[1] Audrey Bienfait,[2] Benjamin Huard,[2] and Alexandre Blais[1,3]

[1]*Institut Quantique and Département de Physique, Université de Sherbrooke, Sherbrooke J1K 2R1 QC, Canada*
[2]*Ecole Normale Supérieure de Lyon, CNRS, Laboratoire de Physique, F-69342 Lyon, France*
[3]*Canadian Institute for Advanced Research, Toronto, M5G1M1 Ontario, Canada*
(Dated: September 20, 2023)

SUPPLEMENTARY NOTE 1 – THEORY

A. Qubit cloaking: detailed derivation

We start from the Lindblad equation

$$\partial_t\hat{\rho} = -\frac{i}{\hbar}[\hat{H},\hat{\rho}] + \kappa\mathcal{D}[\hat{a}]\hat{\rho}, \tag{1}$$

where $\hat{H} = \hat{H}_0 + \hat{H}_1 + \hat{H}_2$, and $\mathcal{D}[\hat{a}]\hat{\rho} = \hat{a}\hat{\rho}\hat{a}^\dagger - (1/2)\{\hat{a}^\dagger\hat{a},\hat{\rho}\}$ is the Lindblad dissipator describing photon decay [1]. We move to the displaced frame $\hat{\rho}_D(t) = \hat{D}^\dagger(\alpha_t)\hat{\rho}(t)\hat{D}(\alpha_t)$ using the displacement operator $\hat{D}(\alpha_t) = \exp\big(\alpha_t\hat{a}^\dagger - \alpha_t^*\hat{a}\big)$. In this frame, the evolution of the system state is governed by the equation

$$\begin{aligned}\partial_t\hat{\rho}_D =& - i[\hat{H}_{\rm tr}/\hbar + \omega_r\hat{a}^\dagger\hat{a} + ig\hat{n}_{\rm tr}(\hat{a}^\dagger - \hat{a}),\hat{\rho}_D]\\ &+ \kappa\mathcal{D}[\hat{a}]\hat{\rho}_D\\ &- i[\mathcal{E}_2(t)\hat{n}_{\rm tr} - ig(\alpha_t - \alpha_t^*)\hat{n}_{\rm tr},\hat{\rho}_D]\\ &+ [\hat{a},\hat{\rho}_D](-\dot{\alpha}_t^* + (i\omega_r - \kappa/2)\alpha_t^* + \mathcal{E}_1(t))\\ &+ [\hat{a}^\dagger,\hat{\rho}_D](-\dot{\alpha}_t - (i\omega_r + \kappa/2)\alpha_t + \mathcal{E}_1(t)),\end{aligned} \tag{2}$$

where we have introduced the transmon Hamiltonian $\hat{H}_{\rm tr} = 4E_C\hat{n}_{\rm tr}^2 - E_J\cos(\hat{\varphi}_{\rm tr})$. The form of the time-dependent complex amplitude α_t is chosen such as to cancel the cavity drive. For this, the last two lines in Supplementary Equation (2) must vanish and we thus enforce

$$\dot{\alpha}_t = -(i\omega_r + \kappa/2)\alpha_t + \mathcal{E}_1(t), \tag{3}$$

or, equivalently,

$$\alpha_t = \alpha_0 e^{-(i\omega_r+\kappa/2)t} + \int_0^t d\tau\, e^{-(i\omega_r+\kappa/2)(t-\tau)}\mathcal{E}_1(\tau), \tag{4}$$

as mentioned in the discussion following Eq.(3) in the main text. In this frame, the cavity drive is then effectively passed to the qubit, see the third line of Supplementary Equation (2). The latter is cancelled if we choose

$$\mathcal{E}_2(t) = -ig(\alpha_t^* - \alpha_t) = -2g{\rm Im}[\alpha_t]. \tag{5}$$

The initial condition α_0 can be set to zero; it has no consequence on cloaking and, in this way, $\mathcal{E}_2(t)$ starts from zero at $t = 0$.

Also worth noting is that accounting for thermal incoherent excitations in the cavity—replacing $\kappa\mathcal{D}[\hat{a}]\hat{\rho}$ by $(\bar{n}_{\rm th} + 1)\kappa\mathcal{D}[\hat{a}]\hat{\rho} + \bar{n}_{\rm th}\kappa\mathcal{D}[\hat{a}^\dagger]\hat{\rho}$ in Supplementary Equation (1), where $\bar{n}_{\rm th}$ is the thermal population—, qubit decay and dephasing, or incorporating the multimode nature of the cavity, does not change the effect or form of the cancellation drive. We come back to incoherent cavity excitation in Sec. K.

For a constant cavity drive amplitude ε_1 turned on at $t = 0$, the cancellation tone takes the form

$$\begin{aligned}\mathcal{E}_2(t) =& g\varepsilon_1\Bigg[\frac{\cos(\omega_1 t + \phi_{1,+}) - \cos(\omega_r t - \phi_+)e^{-\kappa t/2}}{\sqrt{\omega_+^2 + (\kappa/2)^2}}\\ &- \frac{\cos(\omega_1 t - \phi_{1,-}) - \cos(\omega_r t - \phi_-)e^{-\kappa t/2}}{\sqrt{\omega_-^2 + (\kappa/2)^2}}\Bigg],\end{aligned} \tag{6}$$

where $\omega_\pm = \omega_r \pm \omega_1$, $\phi_\pm = \arctan(-2\omega_\pm/\kappa)$, and $\phi_{1,\pm} = \phi_1 + \phi_\pm$. With $\omega_r \gg \kappa$ and taking $\omega_1 \sim \omega_r$, the leading terms are the ones in the second line of Supplementary Equation (6) and the cancellation tone can be approximated by

$$\mathcal{E}_2(t) \approx A_-\big[\cos(\omega_1 t - \phi_{1,-}) - \cos(\omega_r t - \phi_-)e^{-\kappa t/2}\big], \tag{7}$$

with $A_- = -g\varepsilon_1/\sqrt{\omega_-^2 + (\kappa/2)^2}$. In experiments, a cancellation tone ansatz of the form Supplementary Equation (7) can be used where A_-, $\phi_{1,-}$, and ϕ_- are unknown parameters that can be optimized in a Ramsey-like experiment by minimizing the ac-Stark shift and measurement-induced dephasing, see Fig. 3 in the main text. Importantly, the use of the cancellation tone in Supplementary Equation (6) or (7) demands knowledge of the bare cavity frequency ω_r. An incorrect estimate for ω_r will lead

* These authors contributed equally to this work.

to imperfect cancellation, but only for transient times before the terms oscillating at ω_r in Supplementary Equation (6) or (7) become negligible due to the exponential decay $e^{-\kappa t/2}$.

In practice, there can be small crosstalks by which the drive on the cavity (qubit) also weakly drives the qubit (cavity). Formally, for a known $\mathcal{E}_1(t)$, the simple form of $\mathcal{E}_2(t) = -2g\text{Im}[\alpha_t]$ which achieves the cancellation is replaced in this case by a linear Volterra equation of the second kind: $\mathcal{E}_2(t) = f(t) + \int_0^t d\tau K(t,\tau)\mathcal{E}_2(\tau)$ with a separable Kernel $K(t,\tau) = \sum_j g_j(t)h_j(\tau)$. This equation is guaranteed to have a unique solution. In practice, the experimental calibration of the cancellation tone's amplitude and phase deals with this potential crosstalk.

B. Two-states and RWA approximations for the transmon

In the two-level approximation for the transmon and using the rotating wave approximation (RWA), the Hamiltonian $\hat{H}_0 + \hat{H}_1$ becomes

$$\begin{aligned}\hat{H}_0 + \hat{H}_1 =& \frac{\hbar\omega_q}{2}\hat{\sigma}^z + \hbar\omega_r \hat{a}^\dagger\hat{a} + \hbar g'(\hat{\sigma}^+\hat{a} + \hat{\sigma}^-\hat{a}^\dagger) \\ &- \frac{\hbar\varepsilon_1(t)}{2}(\hat{a}^\dagger e^{-i\omega_1 t} + \hat{a}e^{i\omega_1 t}).\end{aligned} \tag{8}$$

In terms of the transmon parameters [2], the qubit frequency is given by $\hbar\omega_q \approx \sqrt{8E_C E_J} - E_C$ and the coupling is $g' \approx (g/2)(E_J/2E_C)^{1/4}$. With these approximations, the cancellation tone on the qubit takes the form

$$\hat{H}_2 = \hbar g'(\text{Re}[\alpha_t]\hat{\sigma}^x - \text{Im}[\alpha_t]\hat{\sigma}^y), \tag{9}$$

with

$$\alpha_t = \frac{i}{2}\int_0^t d\tau\, e^{-(i\omega_r + \kappa/2)(t-\tau)}\varepsilon_1(\tau)e^{-i\omega_1\tau}. \tag{10}$$

To obtain the results of Fig. 2 and Fig. 3a of the main text, we numerically integrate the master equation of Supplementary Equation (1) with the Hamiltonians of Supplementary Equation (8) and Supplementary Equation (9).

C. Cloaking of a general multilevel quantum system

As is made clear by the above two examples, as long as the coupling Hamiltonian is linear in the cavity mode creation and annihilation operators ($\hat{a}$ and $\hat{a}^\dagger$), the approach proposed here is applicable to a wide range of quantum systems where two driving ports couple asymmetrically to the cavity and the quantum system. Indeed, we can generalize our cloaking method for any multilevel quantum system with Hamiltonian $\hat{H}_q$, coupled to a cavity with $\hat{H}_g = g\hat{O}_q(e^{-i\phi}\hat{a}^\dagger + e^{i\phi}\hat{a})$ via any quadrature (i.e. arbitrary phase ϕ), where $\hat{O}_q$ is any operator on the multilevel system.

For this general case, the cancellation term $\hat{H}_2(t)$ in the Lindblad equation $\partial_t\hat{\rho} = -\frac{i}{\hbar}[\hat{H},\hat{\rho}] + \kappa\mathcal{D}[\hat{a}](\hat{\rho})$, where

$$\begin{aligned}\hat{H} =& \hat{H}_q + \hat{H}_g + \hbar\omega_r\hat{a}^\dagger\hat{a} \\ &+ i\hbar\mathcal{E}_1(t)(\hat{a}^\dagger - \hat{a}) + \hat{H}_2(t),\end{aligned} \tag{11}$$

is given by

$$\hat{H}_2(t) = -g(\alpha_t e^{i\phi} + \alpha_t^* e^{-i\phi})\hat{O}_q, \tag{12}$$

with α_t as in Supplementary Equation (4).

D. Inclusion of the Purcell filter

Considering a general multilevel system with Hamiltonian $\hat{H}_q$ as in the previous subsection, we now account for the presence of a Purcell filter cavity coupled to the readout cavity, such that the total Hamiltonian reads

$$\begin{aligned}\hat{H}(t) =& \hat{H}_q + \hat{H}_g + \hbar\omega_r\hat{a}^\dagger\hat{a} + \hat{H}_2(t) \\ &+ \hbar\omega_f\hat{f}^\dagger\hat{f} + \hbar J(\hat{a}^\dagger + \hat{a})(\hat{f}^\dagger + \hat{f}) \\ &+ i\hbar\varepsilon_1(t)\sin(\omega_1 t + \phi_1)(\hat{f}^\dagger - \hat{f}),\end{aligned} \tag{13}$$

where $\hat{f}^{(\dagger)}$ is the annihilation (creation) operator of the Purcell mode of frequency ω_f, and now the drive is on the Purcell cavity instead of the readout cavity.

In the presence of photon loss at the Purcell filter cavity, the master equation takes the form

$$\partial_t\hat{\rho} = -\frac{i}{\hbar}[\hat{H}(t),\hat{\rho}] + \kappa_f\mathcal{D}[\hat{f}]\hat{\rho}. \tag{14}$$

Following the above approach, we now move to a displaced frame $\hat{\rho}_D(t) = \hat{D}_r^\dagger(\alpha_t)\hat{D}_f^\dagger(\beta_t)\hat{\rho}(t)\hat{D}_r(\alpha_t)\hat{D}_f(\beta_t)$, where the subscripts r and f indicate that the displacement operator acts on the Hilbert space of the readout cavity mode $\hat{a}$ or the Purcell filter cavity mode $\hat{f}$, respectively. Moreover, we choose α_t and β_t such that they follow the coupled equations of motion

$$\begin{aligned}\partial_t\alpha_t =& -i\omega_r\alpha_t - iJ(\beta_t^* + \beta_t) \\ \partial_t\beta_t =& -(i\omega_f + \kappa_f/2)\beta_t - iJ(\alpha_t^* + \alpha_t), \\ &+ \varepsilon_1(t)\sin(\omega_1 t + \phi_1).\end{aligned} \tag{15}$$

With this choice, the drive on the Purcell cavity is cancelled and effectively appears on the qubit. This effective drive on the qubit in the displaced frame is cancelled by the Hamiltonian $\hat{H}_2(t)$ in Supplementary Equation (12), with α_t now the solution to Supplementary Equation (15).

E. Gates and imperfect cancellation

Here we report on the simulation of logical gates on the transmon in the presence of a cavity drive. While the gate fidelity can be maintained constant for any cavity drive amplitude if the exact cancellation tone is used, here we explore the deviations that could arise if the cancellation tone is inexact.

As an example, consider the approximate cancellation in Supplementary Equation (7) which is very precise for $\omega_1 \sim \omega_r \gg \kappa$. Among other kinds of errors, here we consider that resulting from using the simpler ansatz

$$\mathcal{E}_2^{\text{ansatz}}(t) = A\cos(\omega_1 t - \phi)(1 - e^{-\kappa t/2}) \tag{16}$$

and study how the gate fidelity deviates from the ideal result.

We consider the Lindblad equation

$$\partial_t \hat{\rho} = -\frac{i}{\hbar}[\hat{H} + \hat{H}_{\text{gate}}, \hat{\rho}] + \kappa \mathcal{D}[\hat{a}]\hat{\rho}, \tag{17}$$

where $\hat{H}$ includes the transmon qubit and cavity Hamiltonian $\hat{H}_0$, the cavity drive $\hat{H}_1$, and the cancellation $\hat{H}_2$ (if present). The gate consists in a π-rotation of $t_{\text{g}} = 25$ ns duration. Using DRAG to eliminate leakage [3], the gate Hamiltonian reads

$$\begin{aligned}\hat{H}_{\text{gate}} =& \hbar\varepsilon_{\text{g}} \left\{ \frac{1}{\cosh[f(t)]} \sin(\tilde{\omega}_q t + \phi_g) \right. \\ &\left. + d_g \frac{\sinh[f(t)]}{\cosh^2[f(t)]} \cos(\tilde{\omega}_q t + \phi_g) \right\} \hat{n}_{\text{tr}}\end{aligned} \tag{18}$$

where $f(t) = \sqrt{\frac{\pi}{2}}(t - t_{\text{g}}/2)/(t_{\text{g}}/4)$, $\tilde{\omega}_q$ the transmon $g-e$ transition frequency dressed by the cavity, and $\{\varepsilon_g, d_g, \phi_g\}$ the set of parameters which are optimized to realize high-fidelity operations. This optimization is performed only once in the absence of cavity drive and cancellation tone. We note that this waveform for the π pulse is the same as the one we use in our experiment.

Numerically, to characterize the gate fidelity, we use [4]

$$\mathcal{F}_{\text{no drives}} = \frac{1}{6}\sum_i \text{Tr}\left[\hat{U}\hat{\rho}_i\hat{U}^\dagger \mathcal{E}_{t_g}(\hat{\rho}_i)\right], \tag{19}$$

which averages over the six cardinal states ($i = \pm x, \pm y, \pm z$) of the Bloch sphere in the dressed computational subspace $\{|\overline{g,0}\rangle, |\overline{e,0}\rangle\}$, corresponding to the qubit logical states dressed by the cavity [5]. The operator $\hat{U}$ is the X Pauli operator in this subspace and $\mathcal{E}_{t_g}$ is the dynamical map which evolves the state according to the master equation of Supplementary Equation (17).

Using $E_J/h = 84.28 E_C/h = 16.826$ GHz, $\omega_r/2\pi = 7.657$ GHz, $g/2\pi = 140.6$ MHz, $\kappa/2\pi = 10.1$ MHz, and accounting for three transmon energy levels, we obtain the optimal values $\varepsilon_g/2\pi \approx 28.51$ MHz, $d_g \approx 0.09$, and $\phi_g/2\pi \approx -0.027$, producing a gate error $E = 1 - \mathcal{F}_{\text{no drives}} \approx 0.003$ in the absence of cavity and cancellation tones. This value corresponds to the coherence limit ($\sim 1 - e^{-\gamma_\kappa t_g/2}$) set by the Purcell decay rate γ_κ [5].

In the presence of the cavity drive and in the displaced frame introduced in the main text and in Supplementary Equation (2), the gate realizes a high-fidelity X π-rotation within the subspace $\{|\overline{g,0}\rangle, |\overline{e,0}\rangle\}$ as long as the qubit is cloaked. Back in the laboratory frame, the dynamical evolution starting with the qubit in an arbitrary state and the cavity in the vacuum state, turning on the cavity drive and the cancellation tone, and performing the logical gate corresponds to the transformation

$$\psi_g |\overline{g,0}\rangle + \psi_e |\overline{e,0}\rangle \to \hat{D}(\alpha_{t_g})(\psi_g |\overline{e,0}\rangle + \psi_e |\overline{g,0}\rangle). \tag{20}$$

To account for the displacement operator, the expression for the gate fidelity in Supplementary Equation (19) in the presence of drive and cancellation is modified to

$$\mathcal{F} = \frac{1}{6}\sum_i \text{Tr}\left[\hat{\sigma}_i \mathcal{E}_{t_g}(\hat{\rho}_i)\right], \tag{21}$$

where

$$\hat{\sigma}_i = \hat{D}(\alpha_{t_g})\hat{U}\hat{\rho}_i\hat{U}^\dagger\hat{D}^\dagger(\alpha_{t_g}). \tag{22}$$

Note that for $\varepsilon_1 = 0$ (and thus $\mathcal{E}_2 = 0$), $\alpha_{t_g} = 0$ and this expression corresponds to Supplementary Equation (19).

In Supplementary Figure 1a we show the average gate error obtained from the above expression and integration of the master equation of Supplementary Equation (17) as a function of ε_1 when the cancellation tone is off (blue line) and with the exact cancellation tone (red line). In the latter case and as expected from the discussion in the main text, the gate error is constant at $\sim 0.3\%$ for all drive amplitudes but rapidly increases with drive amplitude in the absence of cancellation. In Supplementary Figure 1b the gate error is plotted considering the approximate cancellation tone in Supplementary Equation (7), but in the hypothetical case of an imprecise estimate of the value of ω_r ($\neq \omega_r^{\text{exact}}$), highlighting the importance of correctly determining the bare cavity frequency to use in the cancellation tone. We reiterate that the imprecision leads to a slightly incomplete cancellation which is corrected after a short transient due to the exponential decay in time of the driving terms at the bare cavity frequency. When necessary, the bare frequency ω_r could be a parameter to optimize over in the experimental calibration of the cancellation tone. In Supplementary Figure 1c and d, we show the error when we use the cancellation tone in Supplementary Equation (6) but with phase and amplitude relative offsets, respectively. In this case, we replace $\phi_\pm \to \phi_\pm(1 + \delta\phi)$ or $\varepsilon_1 \to \varepsilon_1(1 + \delta\varepsilon)$ in the equation for $\mathcal{E}_2(t)$, taking $\phi_1 = 0$ for simplicity. In the case of phase offset, the main contribution to the deterioration of the gate comes from the offset in ϕ_-, which enters in the leading terms (second line in Supplementary Equation (6)). Finally, in Supplementary Figure 1e, we show the result of using the simpler anzat in Supplementary Equation (16) with a single frequency ω_1, showing that when $\omega_1 \approx \omega_r$ the gate maintains a high fidelity and deteriorates as the cancellation tone frequency shifts away.

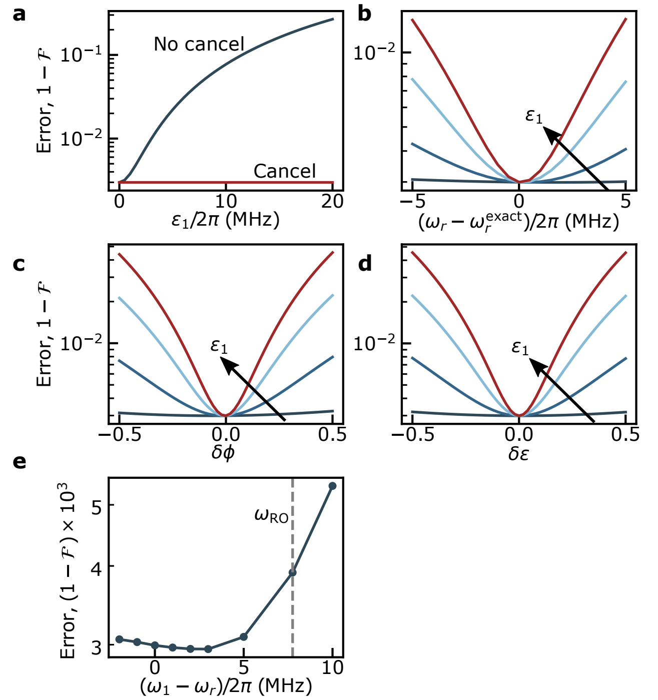

Supplementary Figure 1. Numerical X-gate average error. **a**, Error as a function of cavity drive amplitude without cancellation (blue line) and with exact cancellation (red line). **b**, Error when the cancellation tone in Supplementary Equation (7) is used, but with a hypothetical imprecise estimate of the cavity frequency value, $\omega_r \neq \omega_r^{\rm exact}$. Different colors correspond to $\varepsilon_1/2\pi = 1$, 5, 10, and 15 MHz. The arrow indicates increasing values of ε_1. **c,d**, Error when there is, respectively, a phase ($\delta\phi$) or amplitude ($\delta\varepsilon$) relative offset (see text). Same ε_1 values as in **b**. In all **a,b,c,d**, the cavity is driven at the readout frequency, $\omega_1 = \omega_{\rm RO} = \tilde{\omega}_r + (\chi_g + \chi_e)/2$. **e**, The simpler cancellation tone ansatz in Supplementary Equation (16) is used, which consist of a single frequency ω_1. The drive amplitude is $\varepsilon_1/2\pi = 15\,\text{MHz}$. The vertical dashed line indicates the value of the readout frequency, $\omega_{\rm RO}$. The value of all parameters are indicated in the text.

F. Gate simulation including Purcell cavity

Here we give details about the simulations used to extract the calculated average π-rotation (X gate) error results shown in Fig. 5 in the main text, for the case of a cloaked qubit. Below we also show results for this error when the qubit is not cloaked.

Starting from Supplementary Equations (13) and (14) for the case of the transmon, i.e. $\hat{H}_{\rm q} = \hat{H}_{\rm tr}$ and $\hat{H}_g = ig\hat{n}_{\rm tr}(\hat{a}^\dagger - \hat{a})$, we first diagonalize the Hamiltonians of the readout and Purcell cavities. In this basis, they become hybridized. Since $\omega_r \sim \omega_f \gg |J|$, we use the rotating-wave approximation in the cavity-cavity coupling. Moreover, adding intrinsic transmon decay and dephasing, we arrive at

Parameter	Value
$E_C/2\pi\hbar$	208.09 MHz
$E_J/2\pi\hbar$	16.23 GHz
$\omega_r/2\pi$	7.64744 GHz
$\omega_f/2\pi$	7.63166 GHz
$g/2\pi$	166.85 MHz
$J/2\pi$	26.14 MHz
$\kappa_f/2\pi$	29.1 MHz
$\gamma/2\pi$	6.35 kHz
$\gamma_\phi/2\pi$	18.04 kHz
t_g	25 ns
$\varepsilon_g/2\pi$	29.192 MHz
d_g	0.0869
$\phi_g/2\pi$	-0.0273

Supplementary Table I. Parameters for the numerical simulation of the X gate average error including a Purcell cavity.

$$\begin{aligned}\partial_t\hat{\rho} = & -i[\hat{H}(t) + \hat{H}_{\rm gate}(t), \hat{\rho}] \\ & + \kappa_f \mathcal{D}[\sin\frac{\theta}{2}\hat{a}_+ + \cos\frac{\theta}{2}\hat{a}_-]\hat{\rho} \\ & + \gamma\mathcal{D}[\hat{d}]\hat{\rho} + 2\gamma_\phi\mathcal{D}[\hat{d}^\dagger\hat{d}]\hat{\rho},\end{aligned} \tag{23}$$

with $\hat{H}_{\rm gate}$ given in Supplementary Equation (18), $\hat{d} = \sum_j \sqrt{j}\,|j\rangle\langle j+1|$ is a lowering operator in the energy eigenbasis of the transmon, $\hat{H}_{\rm tr}|j\rangle = E_j|j\rangle$, and

$$\begin{aligned}\hat{H}(t) = & \hat{H}_{\rm tr} + \omega_+\hat{a}_+^\dagger\hat{a}_+ + \omega_-\hat{a}_-^\dagger\hat{a}_- \\ & + ig\hat{n}_{\rm tr}\cos(\theta/2)(\hat{a}_+^\dagger - \hat{a}_+) \\ & - ig\hat{n}_{\rm tr}\sin(\theta/2)(\hat{a}_-^\dagger - \hat{a}_-) \\ & + i\mathcal{E}_1(t)\sin(\theta/2)(\hat{a}_+^\dagger - \hat{a}_+) \\ & + i\mathcal{E}_1(t)\cos(\theta/2)(\hat{a}_-^\dagger - \hat{a}_-),\end{aligned} \tag{24}$$

where

$$\begin{pmatrix}\hat{a}_+ \\ \hat{a}_-\end{pmatrix} = \begin{pmatrix}\cos(\theta/2) & \sin(\theta/2) \\ -\sin(\theta/2) & \cos(\theta/2)\end{pmatrix}\begin{pmatrix}\hat{a} \\ \hat{f}\end{pmatrix}, \tag{25}$$

with $\tan\theta = 2J/(\omega_r - \omega_f)$ and

$$\omega_\pm = \frac{\omega_r + \omega_f}{2} \pm \frac{1}{2}\sqrt{(\omega_r - \omega_f)^2 + 4J^2}. \tag{26}$$

The full set of simulation parameters that we use are shown in Supplementary Table I. With these values of γ and γ_ϕ, we obtain a qubit lifetime and Ramsey coherence time of $T_1 = 25\ \mu$s and $T_2 = 7.5\ \mu$s, respectively, as in our experiment.

For the numerical simulations of the X gate, we work in the displaced frame where the drive on the Purcell cavity is effectively passed to the qubit. This allows us to integrate the master equation with truncated Hilbert spaces for the modes $\hat{a}_\pm$ that are smaller than would be required if we worked in the laboratory frame. In the results shown in Supplementary Figure 2 (and Fig. 5

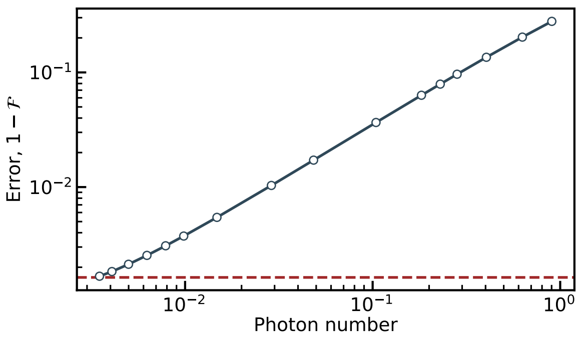

Supplementary Figure 2. Predicted X-gate average error accounting for the Purcell cavity. As a function of the number of photons in the readout cavity at the end of the gate, the blue curve with dots shows the error without cancellation, and the dashed line the error with cancellation. The minimum error attained is $\sim 0.163\%$. Parameters are indicated in Supplementary Table I.

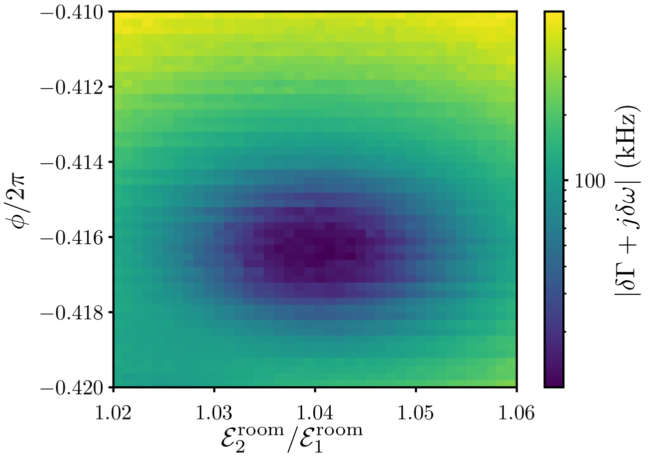

Supplementary Figure 3. Calibration of the cancellation phase ϕ and amplitude $\mathcal{E}_2^{\rm room}$. We minimize the extra-dephasing $\delta\Gamma$ and ac-Stark shift $\delta\omega$ measured by Ramsey interferometry. The cavity drive amplitude is $\varepsilon_1/2\pi = 107\,$MHz.

in the main text), we use the definition of Supplementary Equation (21), where the six cardinal states of the Bloch sphere are now those of the logical subspace $\{|\overline{g,0_+,0_-}\rangle, |\overline{e,0_+,0_-}\rangle\}$, corresponding to the ground and excited states of the qubit dressed by the hybridized modes $\hat{a}_\pm$ (here $0_\pm$ is the zero Fock state of these modes).

The gate error obtained from numerical simulations quantitatively agree with experiments in the presence of cloaking (see dashed red line in Fig. 5 in the main text and Supplementary Figure 2). In the absence of cloaking, full blue line in Supplementary Figure 2, we find numerically the expected increase of the gate error with cavity photon number but the agreement with the experimental observations is not quantitative. While the average gate error in the presence of cloaking is coherence limited and thus simple to fit to the experimental results by adjusting γ and γ_ϕ, in the absence of cloaking the error depends on the quality of the fit of the bare parameters used in the model, as well as on a precise calibration of the experimental attenuation of drive power. An accurate comparison of numerical results to experiment data as a function of photon number is therefore challenging. For this reason, we do not show the numerical results together with the experimental data in Fig. 5 of the main text.

SUPPLEMENTARY NOTE 2 – EXPERIMENT

G. Calibration of the cancellation tone

The cancellation drive amplitude and phase is calibrated using Ramsey interferometry where the drives $\mathcal{E}_{1/2}$ are applied between the two $\pi/2$ pulses of the Ramsey sequence. For a given drive $\mathcal{E}_1$ on port 1, we minimize the extra-dephasing and ac-Stark shift $|\delta\Gamma + i\delta\omega|$, see Supplementary Figure 3. We used a cancellation tone ansatz of the form given in Supplementary Equation (7).

The Ramsey sequence (with a constant ε_1) is not the most sensitive way to measure a miscalibration of the cancellation tone in the transient regime. For example, with $\kappa/2\pi = 10\,$MHz in the experiment reported here, the steady state of the cavity field is reached in $\sim 100\,$ns, which is small in comparison to the tens of microseconds of the employed Ramsey sequence. If further precision at early times is necessary, only two more calibration steps are needed to calibrate the time-delay mismatch and phase between the drive term at ω_1 and the term at ω_r in Supplementary Equation (7). These calibrations can be done separately from the previous calibrations and do not influence the already calibrated parameters.

Nevertheless, as shown in Supplementary Figure 1e, corresponding to the worst-case scenario where the cancellation-tone term at ω_r is missed altogether (c.f. Supplementary Equation (16)), only a small error in the X gate is introduced.

H. Histograms and overlap error

Each heterodyne signal is demodulated over an integration time $t_{\rm int}$ resulting in a complex value α. Repeating this measurement, we obtain probability distributions of the complex amplitudes $P_{g,e}(\alpha, t_{\rm int})$ when the qubit has been prepared in state $|g\rangle$ or $|e\rangle$. Typical probability distributions are shown in Supplementary Figure 4 for $t_{\rm int} = 196\,$ns in case of standard dispersive or arm-and-release readout. The measured histograms reveal two Gaussian distributions corresponding to each qubit state. Some readout amplitudes α fall out of these two distributions, which happens with a probability $p_{\rm out} = 0.07\%$, likely due to transmon ionization [6]. The readout amplitude has been chosen on the onset of ionization in order to

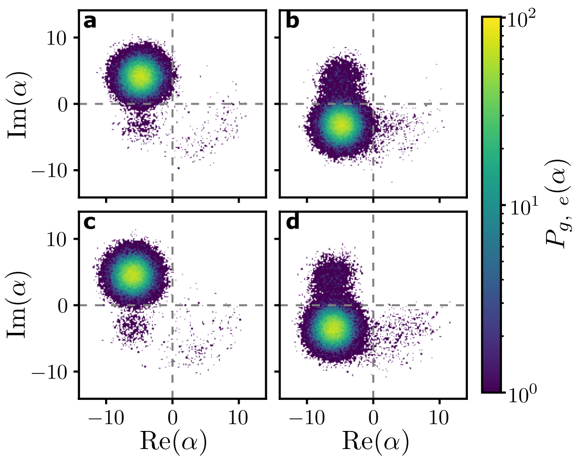

Supplementary Figure 4. Measured Husimi distributions for standard dispersive and arm-and-release readout. Histograms of the demodulated heterodyne signal for $t_{\rm int} = 196\,\mathrm{ns}$ and $\varepsilon_1/2\pi = 63.7\,\mathrm{MHz}$ for the standard dispersive readout (**a** and **b**) and for the arm-and-release readout (**c** and **d**) when the qubit is prepared in its ground state (**a** and **c**) or in its excited state (**b** and **d**).

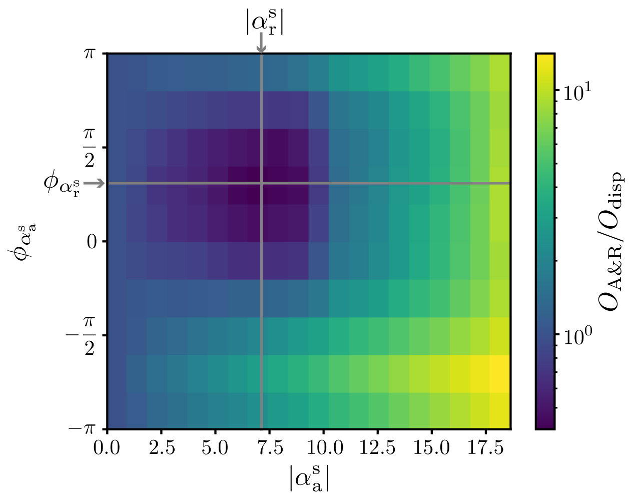

Supplementary Figure 5. Readout overlap error comparison for standard disperive and arm-and-release methods. Error ratio $O_{\rm A\&R}/O_{\rm disp}$ between arm-and-release $O_{\rm A\&R}$ and standard dispersive $O_{\rm disp}$ readout errors as function of arming amplitude $|\alpha^{\rm s}_{\rm a}|$ and phase $\phi_{\alpha^{\rm s}_{\rm a}}$. The overlap errors are extracted for a 140 ns integration time.

reach a good trade off between the error of finite separation between pointer states and the error $p_{\rm out}$ of ionizing the transmon.

From these histograms $P_{g,e}(\alpha, t_{\rm int})$, we compute the overlap (their normalized 2D scalar product)

$$O(t_{\rm int}) = \frac{\int P_g(\alpha,t_{\rm int})P_e(\alpha,t_{\rm int})d\alpha}{\sqrt{\int P_g(\alpha,t_{\rm int})^2 d\alpha}\sqrt{\int P_e(\alpha,t_{\rm int})^2 d\alpha}}. \quad (27)$$

To compute the average fidelity $\mathcal{F} = 1 - [P(g|e) + P(e|g)]/2$ with $P(x|y)$ the probability to measure x when state y was prepared, we project the dataset onto the imaginary axis. Defining the threshold $q_{\rm th} = 0$, we compute the error probabilities $P(g|e) = P(\mathrm{Im}(\alpha) > q_{\rm th}|e) = (1.07 \pm 0.14)\,\%$ and $P(e|g) = P(\mathrm{Im}(\alpha) \leq q_{\rm th}|g) = (0.23 \pm 0.14)\,\%$. We thus obtain $\mathcal{F} = (99.35 \pm 0.14)\,\%$. The uncertainty $\pm 0.14\,\%$ comes from the finite number $N = 10^6$ of repetitions. The error of $0.65\,\%$ in the average infidelity is mostly explained by the following processes. By fitting the distribution with double Gaussians [7], we compute the error due to finite separation ($\sim 0.13\%$) between the two Gaussians corresponding to the two states of the qubit. In these experiments, the qubit is first prepared in the ground state $|g\rangle$ using measurement-based feedback with the usual dispersive readout. Using a second standard dispersive readout, we estimate the error of wrong preparation in the ground state before the arming step as $P_{\rm disp}(e|g) \sim 0.2\%$. Incorrect excited state preparation is also explained by imperfect π pulse due to the coherence limit of the qubit (giving in average, an error of $\sim 0.08\%$, see Fig. 5 of the main text). Error due to relaxation during the measurement correspond to $\sim \frac{1}{2}(1 - e^{-t_{\rm int}/2T_1}) \sim 0.2\%$.

I. Pre-arming amplitude and phase optimization

Because the cavity does not respond at the same frequency during the arming and release steps, the amplitude and phase of the arming step needs to be optimized to obtain the fastest separation between cavity states during readout. Indeed, during the arming step, the steady-state amplitude is given by $\alpha^{\rm s}_{\rm a} = (\varepsilon_{1,a}e^{i\phi_{1,a}}/2)/(\omega_r - \omega_1 - i\kappa/2)$ while in the release step, it is given by $\alpha^{\rm s}_{\rm r,i} = (\varepsilon_{1,r}e^{i\phi_{1,r}}/2)/(\tilde{\omega}_r + \chi_i - \omega_1 - i\kappa/2)$ and depends on the state i of the qubit. For our parameters and choosing $\omega_1 = \tilde{\omega}_r + (\chi_g + \chi_e)/2$, driving at the same amplitude and phase leads to an amplitude ratio of $|\alpha^{\rm s}_{\rm a}|/|\alpha^{\rm s}_{\rm r}| = 0.58$ and phase difference $\phi_{\alpha^{\rm s}_{\rm a}} - \phi_{\alpha^{\rm s}_{\rm r}} = 0.154 \times 2\pi$ where $\phi_{\alpha^{\rm s}_{\rm r}} = (\phi_{\alpha^{\rm s}_{\rm r,g}} + \phi_{\alpha^{\rm s}_{\rm r,e}})/2$ is the average phase obtained for the two states of the qubit.

To optimize the readout for the arm-and-release protocol, we measure the overlap error ratio $O_{\rm A\&R}/O_{\rm disp}$ between the arm-and-release $O_{\rm A\&R}$ and the standard dispersive $O_{\rm disp}$ readouts (Supplementary Figure 5). The chosen arming amplitude and phase is the one minimizing the error ratio $O_{\rm A\&R}/O_{\rm disp}$.

J. Randomized benchmarking under cavity drives

The gate errors under drives in Fig. 5 of the main text are estimated using randomized benchmarking [8]. For that purpose, we compare the fidelity of different pulse sequences (Supplementary Figure 6a). In a reference pulse sequence, a number N_C of random Clifford gates is applied, followed by a recovery gate before reading out

the qubit state. The sequence fidelity $\mathcal{F}_S$ is fitted using $\mathcal{F}_S = Ap_{G,\mathrm{ref}}^{N_C} + B$ from which we extract $p_{G,\mathrm{ref}}$. The same fitting procedure is applied for an interleaved sequence where the gate under test is interleaved with the random gates, resulting in the probability p_G (see Supplementary Figure 6b). The average gate error is then extracted as $\epsilon_X = \frac{1}{2}(1 - \frac{p_G}{p_{G,\mathrm{ref}}})$.

K. Sample and measurement setup

The large features of the sample are made by optical lithography on a Tantalum thin film on a Sapphire substrate, while the Josephson junction of the transmon qubit is fabricated via electronic lithography followed by angle deposition of Al/AlOx/Al in a Plassys evaporator. The readout mode is a $\lambda/4$ coplanar waveguide resonator. The Purcell filter is also a $\lambda/4$ coplanar waveguide resonator, inductively coupled to the readout mode, and is used as a bandpass filter around the readout frequency.

The sample is cooled down to 10 mK in a dilution refrigerator. The diagram of the microwave wiring is given in Supplementary Figure 7. The qubit, readout and cancellation pulses are generated by modulation of continuous microwave tones produced respectively by generators Anapico APSIN20G and Anapico APSIN12G (readout and cancellation tones use the same local oscillator). They are modulated via IQ-mixers where the intermediate frequency (a few tens of MHz) modulation pulses are generated by 6 channels of an OPX from Quantum Machines with a sample rate of 1 GS/s. The acquisition is performed, after down-conversion by its local oscillator, by digitizing the 100 MHz signal with the 1 GS/s ADC of the OPX. The qubit and cancellation pulses are multiplexed into a single transmission line using a diplexer at the lowest temperature stage.

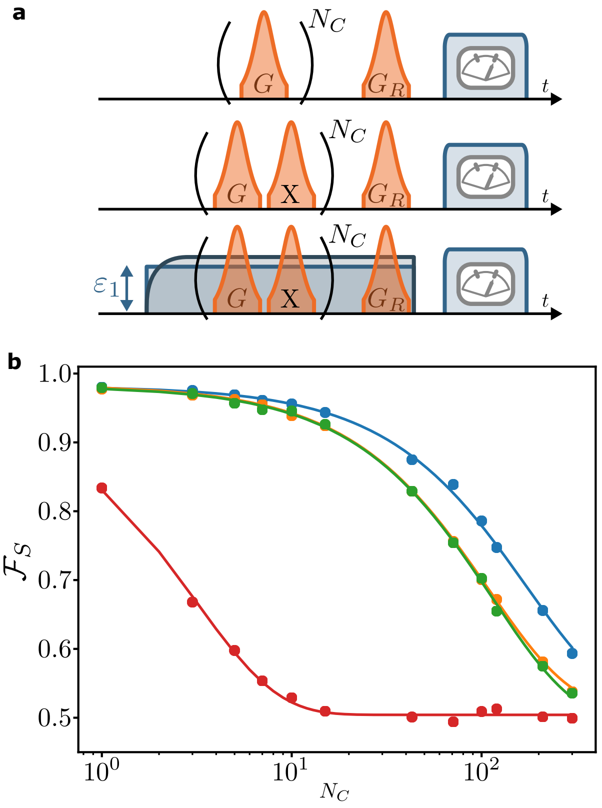

Supplementary Figure 6. X gate randomized benchmarking. **a**, Pulse sequences used for the randomized benchmarking leading to Fig. 5 of the main text: Top, reference sequence; middle, interleaved sequence; bottom, interleaved sequence under arming drives. **b**, Dots: measured sequence fidelity (probability to end in the same state as the initial one) as a function of circuit depth N_C. Lines: fits using $\mathcal{F}_S = Ap_G^{N_C} + B$. Blue: reference sequence, orange: interleaved without drives, red: interleaved with $\mathcal{E}_1$ drive only, and green: interleaved with $\mathcal{E}_1$ and $\mathcal{E}_2$ drives. The drive $\varepsilon_1/2\pi = 6.4$ MHz corresponds to 0.37 photons on average without cancellation and 0.14 photons with cancellation.

SUPPLEMENTARY NOTE 3 – POTENTIAL LIMITATIONS

L. The need of two driving ports

As previously indicated, qubit cloaking necessitates distinct driving ports—one to address the nonlinear mode and another to address the cavity. In the case of circuit QED devices, this requirement is typically fulfilled with the presence of a port for logical operation on the qubit and a second port for readout on the measurement cavity. In other cavity-based platforms, a single driving port may be easily accessible. With distinct driving ports, a possible limitation is that the optimization—in terms of attenuation of thermal noise, dynamical range, or filtering—of the input transmission line for its use in driving the qubit might compete with its optimization for its use for cloaking. This is particularly true because of the requirement to drive the qubit port at the cavity frequency, and relatively large drive amplitudes necessary for cloaking.

M. Qubit cloaking in the ultrastrong coupling regime

In the ultrastrong coupling regime, where the qubit-cavity coupling is comparable to the cavity and qubit transition frequencies, the dissipator no longer assumes the form in Supplementary Equation (1) but rather takes a correlated qubit-cavity form [9, 10]. The assumption of separate dissipation channels for the qubit and cavity is no longer a good approximation and the cloaking drive on the qubit cannot cancel the incoherent effects induced by this correlated dissipation.

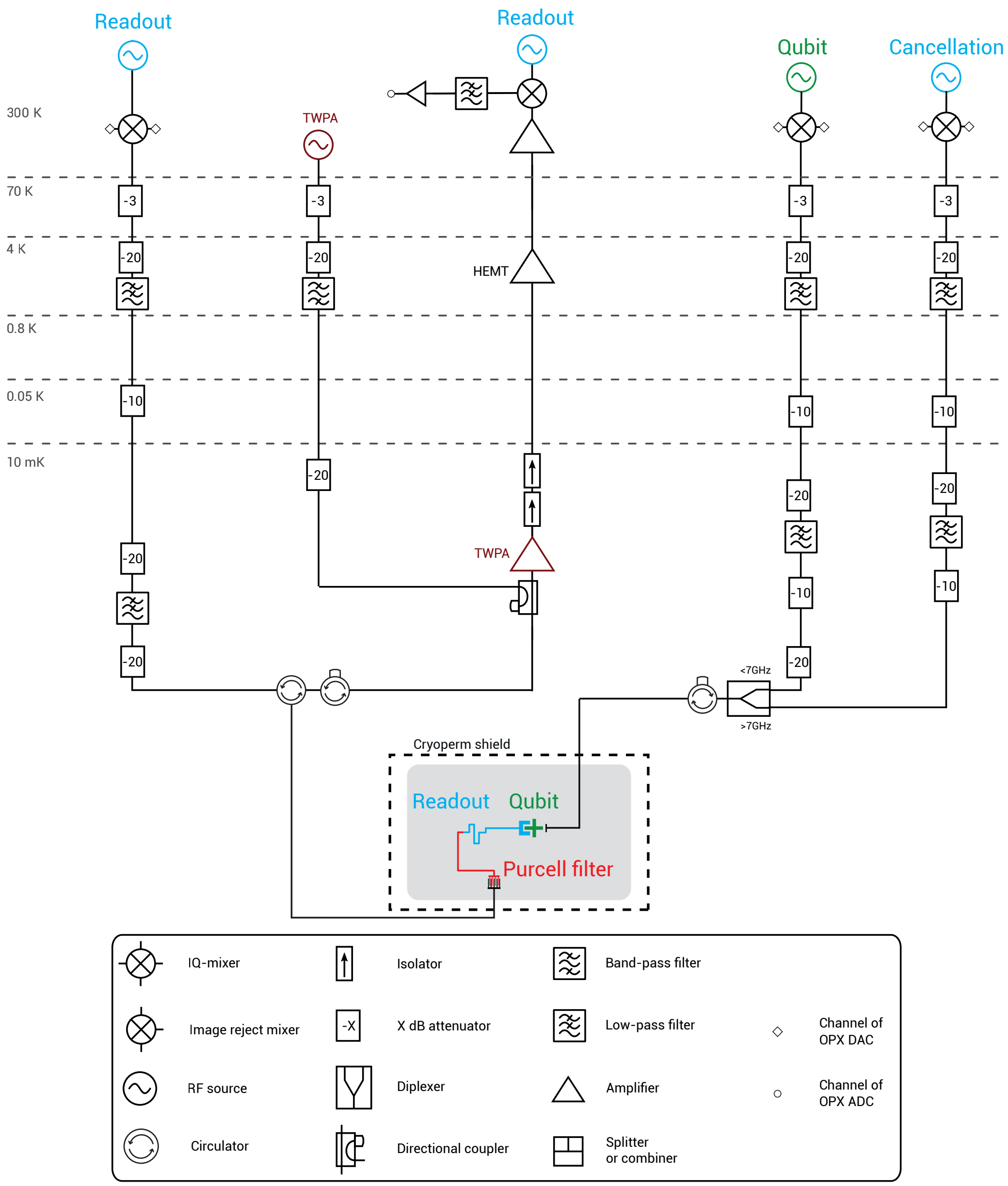

Supplementary Figure 7. Schematic of the microwave setup.

N. The qubit cannot be protected from the thermal excitations in the cavity

While the qubit can be cloaked from the coherent drive on the cavity even in the presence of thermal excitations in the cavity, it cannot be cloaked from the cavity thermal excitations themselves: a coherent tone cannot cloak an incoherent excitation.

[1] H.-P. Breuer and F. Petruccione, *The Theory of Open Quantum Systems* (Oxford University Press, 2007).

[2] J. Koch, T. M. Yu, J. Gambetta, A. A. Houck, D. I. Schuster, J. Majer, A. Blais, M. H. Devoret, S. M. Girvin, and R. J. Schoelkopf, Charge-insensitive qubit design derived from the Cooper pair box, Phys. Rev. A **76**, 042319 (2007).

[3] F. Motzoi, J. M. Gambetta, P. Rebentrost, and F. K. Wilhelm, Simple pulses for elimination of leakage in weakly nonlinear qubits, Phys. Rev. Lett. **103**, 110501

(2009).
[4] M. D. Bowdrey, D. K. Oi, A. J. Short, K. Banaszek, and J. A. Jones, Fidelity of single qubit maps, Phys. Lett. A **294**, 258 (2002).
[5] A. Blais, A. L. Grimsmo, S. M. Girvin, and A. Wallraff, Circuit quantum electrodynamics, Rev. Mod. Phys. **93**, 025005 (2021).
[6] R. Shillito, A. Petrescu, J. Cohen, J. Beall, M. Hauru, M. Ganahl, A. G. Lewis, G. Vidal, and A. Blais, Dynamics of transmon ionization, Phys. Rev. Applied **18**, 034031 (2022).
[7] T. Walter, P. Kurpiers, S. Gasparinetti, P. Magnard, A. Potočnik, Y. Salathé, M. Pechal, M. Mondal, M. Oppliger, C. Eichler, and A. Wallraff, Rapid high-fidelity single-shot dispersive readout of superconducting qubits, Phys. Rev. Applied **7**, 054020 (2017).
[8] E. Knill, D. Leibfried, R. Reichle, J. Britton, R. B. Blakestad, J. D. Jost, C. Langer, R. Ozeri, S. Seidelin, and D. J. Wineland, Randomized benchmarking of quantum gates, Phys. Rev. A **77**, 012307 (2008).
[9] Ángel Rivas, A. D. K. Plato, S. F. Huelga, and M. B. Plenio, Markovian master equations: a critical study, New J. Phys. **12**, 113032 (2010).
[10] F. Beaudoin, J. M. Gambetta, and A. Blais, Dissipation and ultrastrong coupling in circuit QED, Phys. Rev. A **84**, 043832 (2011).
